# Response of soil bacterial community to alpine wetland degradation in arid Central Asia

**DOI:** 10.3389/fpls.2022.990597

**Published:** 2023-01-04

**Authors:** Maidinuer Abulaizi, Mo Chen, Zailei Yang, Yang Hu, Xinping Zhu, Hongtao Jia

**Affiliations:** ^1^ College of Resources and Environment, Xinjiang Agricultural University, Urumqi, China; ^2^ College of Grassland Science, Xinjiang Agricultural University, Urumqi, China; ^3^ Xinjiang Key Laboratory of Soil and Plant Ecological Processes, Urumqi, China

**Keywords:** degraded wetland, degradation biomarker, bacterial diversity, community structure, bacterial function

## Abstract

A large number of studies have reported the importance of bacterial communities in ecosystems and their responses to soil degradation, but the response mechanism in arid alpine wetlands is still unclear. Here, the non-degraded (ND), slightly degraded (SD), and heavily degraded (HD) regions of Bayinbuluk alpine wetland were used to analyzed the diversity, structure and function of bacterial communities in three degraded wetlands using 16S rRNA. The results showed that with the increase of degradation degree, the content of soil moisture (SM) and available nitrogen (AN) decreased significantly, plant species richness and total vegetation coverage decreased significantly, Cyperaceae (Cy) coverage decreased significantly, and Gramineae (Gr) coverage increased significantly. Degradation did not significantly affect the diversity of the bacterial community, but changed the relative abundance of the community structure. Degradation significantly increased the relative abundance of Actinobacteria (ND: 3.95%; SD: 7.27%; HD: 23.97%) and Gemmatimonadetes (ND: 0.39%; SD: 2.17%; HD: 10.78%), while significantly reducing the relative abundance of Chloroflexi (ND: 13.92%; SD: 8.68%; HD: 3.55%) and Nitrospirae (ND: 6.18%; SD: 0.45%; HD: 2.32%). Degradation significantly reduced some of the potential functions in the bacterial community associated with the carbon (C), nitrogen (N) and sulfur (S) cycles, such as hydrocarbon degradation (ND: 25.00%; SD: 1.74%; HD: 6.59%), such as aerobic ammonia oxidation (ND: 5.96%; SD: 22.82%; HD: 4.55%), and dark sulfide oxidation (ND: 32.68%; SD: 0.37%; HD: 0.28%). Distance-based redundancy analysis (db-RDA) results showed that the bacteria community was significantly related to the TC (total carbon) and Gr (*P* < 0.05). The results of linear discriminant analysis effect size (LEfSe) analysis indicate significant enrichments of Alphaproteobacteria and *Sphingomonas* in the HD area. The vegetation communities and soil nutrients changed significantly with increasing soil degradation levels, and *Sphingomonas* could be used as potential biomarker of degraded alpine wetlands.

## Introduction

Although the land surface area of wetland stations is less than one-tenth (Mitsch and Gosselink, 2007), their C storage per unit area accounts more than any other ecosystem (Yarwood, 2018). Wetlands are vital to the preservation of biodiversity and ecological functioning in natural resources (Xia et al., 2015; Wu et al., 2021a). Compared with other ecosystems, alpine wetland ecosystems have relatively singular species, fragile system stability, an extremely sensitive response to human disturbance, and are highly prone to irreversible regional degradation (Chen et al., 2021). Wetland degradation refers to the process of structural changes and functional imbalances of wetland ecosystems under the interference of nature, anthropogenic processes or both (Coban et al., 2022). The degradation of alpine wetlands is mainly caused by climate change, while overgrazing will aggravate the degradation process (Fawzy et al., 2020). Despite the significance of wetlands within the land ecosystem is widely recognized, human factors (e.g., population growth, excessive reclamation, grazing) (Bai et al., 2013) have caused the wetland degradation situation to become increasingly severe and have therefore become an important issue worldwide (Wei and Wu, 2021).

Soil is a complex biological system that serves as a habitat for biogeochemical circulation and enables plant production (Philippot et al., 2013). Soil bacteria is an important driver of the nutrient cycle and energy flow in the ecosystem, and is sensitive to environmental change (Wardle et al., 2004; Peralta et al., 2013; Wagg et al., 2014), thus playing a crucial role in the wetland soil ecosystems (Wu et al., 2021a). Different microbes play different roles in ecosystem environments, such as participating within the C, N, and P cycles, the degradation of cellulose (Xiong et al., 2015), and the production of antibiotics (Chen et al., 2020). The results of many related studies have shown that significant changes in plant biomass, community composition, soil physicochemical properties are the objective manifestations of wetland degradation (Wu et al., 2014; Wang et al., 2015; Chu et al., 2016), as well as important factors that lead to microbial community changes. Soil pH has been shown to be a key factor driving bacterial community diversity and community structure (Wang et al., 2020). Wu et al. (2021a) found that wetland degradation increased the relative abundance of Chloroflexi and Gematimonadetes and significantly reduced the relative abundance of Proteobacteria; However, there were no significant differences in alpha diversity in bacterial communities in different degrees of degraded wetland. In addition, some studies have shown that with the aggravation of degradation, the sulfate respiration process in wetland bacterial community gradually weakens (Liu et al., 2022). The changes in diversity and composition of soil microbial communities reflect the response strategy of microorganisms to adapt to change (Marschner et al., 2003; Segata et al., 2011). The influence mechanism of degradation on alpine grassland was studied from the perspective of plant-soil-microorganism (Zhou et al., 2019). Whether the soil microbial community diversity and composition changes can be used as an indicator of the health or recovery of degraded grassland (Wu et al., 2021b). Li et al. (2022) reported that the degradation of the alpine wetland inhibited the growth of nitrogen-fixing bacteria, leading to the decline of their nitrogen-fixing function. However, there are few reports on the co-change of soil microbial community characteristics and soil environmental factors in different degraded areas of arid alpine wetland under the background of global change. At the same time, this study is the first demonstration of soil bacterial community in different degraded area of Bayinbuluke alpine wetland.

Bayinbuluke alpine wetland is a typical wetland in arid areas of Central Asia, and the only national swan nature reserve in China, which has great ecological strategic value in protecting water resources and biodiversity in this area (Liu et al., 2019; Hu et al., 2022). In the past 20 years, the increasing human disturbance has degraded the Bayinbuluke alpine wetland to varying degrees, which has a profound impact on the plant community and soil nutrient cycle in this area (Zhao et al., 2021). High-cover grassland area decreasing the most, and the number of landscape type patches to low-cover grassland decreased, the landscape fragmentation index of dry land and high-cover grassland was reduced (Shi et al., 2015). Swampy soil with high coverage evolved into sandy land that lead to the loss of soil C (Zhu et al., 2019). Therefore, it is of great significance to study the response mechanism of soil microbial community to the degradation of alpine wetlands in arid areas of Central Asia, for better understanding the wetland ecosystem process under the condition of global environmental change, and providing reference datum for the restoration with sustainable use of degraded alpine wetlands. For this we used high-throughput sequencing technology to analyze the different soil bacterial community characteristics in areas within the Bayinbuluk alpine wetland that have been subjected to different degrees of degradation. The aims of the research are to 1) explore the changing characteristics of the plant community and soil physicochemical properties in wetlands with different degrees of degradation, as well as their relationship to the change of soil bacterial community composition; and 2) elucidate discrepancies in bacterial community structure, and identify microbes that could be employed as alpine wetland degradation biomarkers; 3) to reveal the effects of degradation on the diversity, structure and function of soil bacterial community in alpine wetland.

## Materials and methods

### Study area information

**Figure 1:**
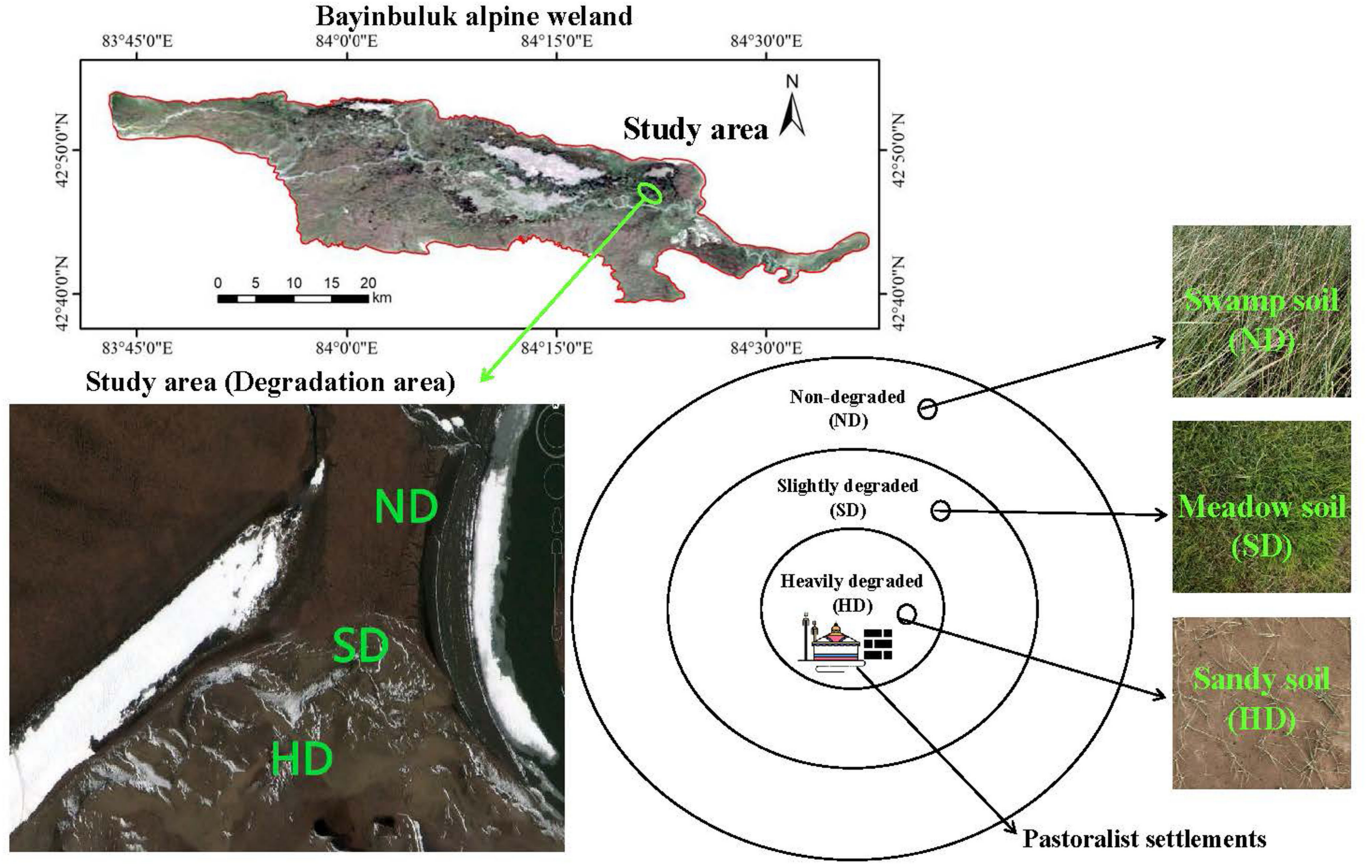
General overview of the non-degraded (ND), slightly degraded (SD), and heavily degraded (HD) alpine wetlands.

This research was conducted in the Bayinbuluk National Nature Reserve (E 82°59′-83°31′, N 42°45′-43°00′) in Xinjiang, China, in August 2019. The Bayinbuluk alpine wetland covers an area of over 770 km^2^, with an altitude of 2300-3042 m. The annual average temperature and precipitation in the area are approximately -4.6 °C, 273 mm, respectively. The water source is snow, ice meltwater, and underground diving overflow, and the underground water level is 0.5-1.0 m deep. The Bayinbuluk alpine wetland has been degraded in different degrees in recent years due to the influence of natural and human disturbance factors, such as climate warming and grazing. The study area was divided into three degraded areas with different degrees: ND, SD and HD, according to aboveground plant biomass, total vegetation coverage, and vegetation coverage of Cyperaceae and Gramineae (Zhou et al., 2019). **Figure 1** and **Table 1** summarizes the overall situation of wetland degradation in the area.

**Table 1:** General information of the non-degraded (ND), slightly degraded (SD) and heavily degraded (HD) alpine wetland areas.

	ND	SD	HD
**Plant coverage**	85.00%	70.00%	15.00%
**Plant height**	48.60 cm	8.20 cm	5.50 cm
**Aboveground biomass**	107.33 ± 23.92 g/m^2^	55.73 ± 3.23 g/m^2^	10.20 ± 0.83 g/m^2^
**Soil type**	Swamp	swamp meadow	sandy
**Grazing intensity**	0.65 head/hm^2^	2.09 head/hm^2^	4.15 head/hm^2^
**Dominant plant groups**	*Carex tristachya; Potentilla serices*	*Festuca ovina*	*Leymus*

### Plant measurement and soil sampling

Three large quadrats (50 × 50 m, distance >100 m) were set in the ND, SD, and HD areas, and then randomly selected three small quadrats (1 × 1 m, distance >10 m) in each large quadrat. The characteristics of plant communities in each quadrat were investigated, including plant coverage, species, and height. Aboveground plants were removed after measuring the plant community. A five-point method (The center point of the square and four points on the diagonal that are the same distance from the center point, distance > 0.5m) was used to collect surface fresh bulk soil (0-10 cm) samples using sterile soil sampler. The composite soil samples obtained from each quadrat were mixed evenly, sieved with a 2 mm mesh size (roots and stones were removed) and divided into four parts on average. Three parts (one for use, two for preparation) were used for measuring the soil physicochemical properties (Air-dried soil samples under natural conditions), and the remaining part was used for high-throughput sequencing (stored at -80°C).

### Vegetation characteristics

The aboveground plant biomass is the aboveground plants after drying treatment (30 minutes at 105°C and 48 hours at 65°C). Some samples were ground into fine powder using a freezing ball mill (PM100, Retsch, Germany) sieved with 60 mesh size and total carbon (TC) content was determined by using an element analyzer (Vario El III, Elemental, Germany).

### Soil physicochemical properties

A pH meter (FE28-Standard, Switzerland) was used to measure the soil pH (soil-to-water ratio of 1:5). An element analyzer (Vario El III, Elemental, Germany) was used to determine the TC and nitrogen (TN) concentrations in the soil (Chen et al., 2021), and the soil available nitrogen (AN) was measured using the alkaline hydrolysis diffusion method (Lu, 2000). A spectrophotometer (UV-1780, Japan) was used to assess total (TP) and available phosphorus (AP) with NaHCO_3_ Extraction-Mo-Sb colorimetry (Lu, 2000). Flame photometry (M420, Sherwood, British) was used to estimate total potassium (TK) and available potassium (AK) with NaOH melting method-flame photometer method (Hu et al., 2022). The gravimetric method was used to determine the soil moisture (SM). Bulk density (BD) was determined by the cutting-ring method, which was calculated with the following formula: dry soil weight (g)/soil volume (cm^3^) = BD (g cm^-3^) (Zhou et al., 2019).

### DNA extraction and sequencing

The bacterial community was evaluated by high-throughput sequencing of 16S rRNA gene. Soil total community DNA was extracted with the Power Soil DNA Isolation Kit (MO BIO, USA). The DNA quality and quantity was determined using NanoDrop™ One (Thermo Fisher, USA). Using the primer pair 338F(5’-ACTCCTAGGGAGCA-3’)/806R (5’-GGACTCHVGGGTWTTAT-3’) (Christian et al., 2013). The sequencing and bioinformatics services of all of the samples were completed on the Illumina Hiseq 2500 platform of BMK Cloud (www.biocloud.net, Biomarker Technologies Co. Ltd., Beijing, China) (Chen et al., 2021). SRA (Sequence Read Archive) records will be accessible with the following link after the indicated release date: https://www.ncbi.nlm.nih.gov/sra/PRJNA813909.

### Processing of sequencing data

Flash software (version 1.2.11) and Trimmomatic (version 0.33) software were used to obtain high-quality reads. UCHIME (version 8.1) software was used to remove chimera sequence and obtain the final valid data. USEARCH (version 10.0) software was used to cluster the reads (at a similarity level of 97%) to obtain the operational taxonomic units (OTUs). OTUs with polar abundance (abundance less than 0.005%) were removed, and the OTUs were taxonomically annotated based on the 16S bacteria taxonomy database (Silva, release 132).

### Data analyses

R (version 4.0.2) was used to perform the univariate and multivariate analyses. Analysis of the variance (ANOVA) and the *post-hoc* test least significant difference test (LSD) were used to test significant differences among different areas using the agricolae package (version 0.11.3) (Mendiburu et al., 2016). Car software package (version 0.1-0) and dplyr software package (version 1.0.10) are used normality and homogeneity of variance were checked. The ggalluvial package (version 0.11.3) was used to draw the stack histogram of the bacterial relative abundances (Aime, 2021). The microeco package (version 0.7.1) (Liu et al., 2021) was used to perform the linear discriminant analysis effect size (LEfSe, from phylum to genus level) in different degradation areas. The bacterial community alpha-diversity indices (with rarefied data) and rarefaction curves were determined using Mothur (version 1.30). Functional prediction of soil bacterial communities under different degradation area was done using the FAPROTAX database (Hu et al., 2022).

We have done the PCoA, the ANOSIM and the RDA with rarefied data. The vegan software package (version 2.5.6) was used to construct a PCoA (principal co-ordinate analysis) based on the Bray-Curtis distance to measure the β-diversity of the bacterial community, and the ANOSIM (analysis of similarities) analysis was used to further test whether there were significant differences in different degrees of degradation. The ggvegan software package (version 0.1-0) was used to construct a db-RDA to analyze the relationship between bacterial community structure and environmental factors. Linket software package (version 0.0.2.9) was used to analyze the Pearson correlation and spearman correlation (Huang, 2021).

## Results

### Characteristics of alpine wetlands plant communities under different degradation degrees

In this study, the plant characteristics of different degraded areas in alpine wetlands were different (**Table 2**). In the ND area, species richness was significantly higher than SD and HD areas. Cyperaceae coverage significantly decreased with increasing degradation level, whereas Gramineae coverage increased. *Leymus* was the only gramineous plant found in the HD area (**Table 1**). The coverage of Cyperaceae plants in the ND area was the highest, reaching 80.67 ± 1.67%, which was significantly higher than that in SD (54.67 ± 2.40%) and HD (0.00%) areas. The TC content of plants decreased with the aggravation of degradation.

**Table 2:** Plant communities features of the non-degraded (ND), slightly degraded (SD) and heavily degraded (HD) alpine wetland areas.

	ND	SD	HD
**Plant species richness**	12.00 ± 1.53^a^	8.00 ± 1.15^b^	1.00 ± 0.00^c^
**Plant coverage (%)**	85.33 ± 0.88^a^	70.67 ± 0.67^b^	15.00 ± 0.57^c^
**Gramineae (%)**	4.66 ± 0.33^c^	9.33 ± 0.88^b^	15.00 ± 0.57^a^
**Cyperaceae (%)**	80.67 ± 1.67^a^	54.67 ± 2.40^b^	0.00 ± 0.00^c^
**Forb (%)**	3.59 ± 0.20^b^	7.21 ± 0.15^a^	0.00 ± 0.00^c^
**Plant total carbon (g kg^-1^)**	424.37 ± 1.11^ab^	427.11 ± 10.37^a^	410.17 ± 9.10^b^

mean ± standard error (n = 3). The same lowercase letter indicates no significant difference, but the difference is significant at the 0.05 level.

### Differences in soil physicochemical properties of alpine wetlands under different degradation levels

**Figure 2:**
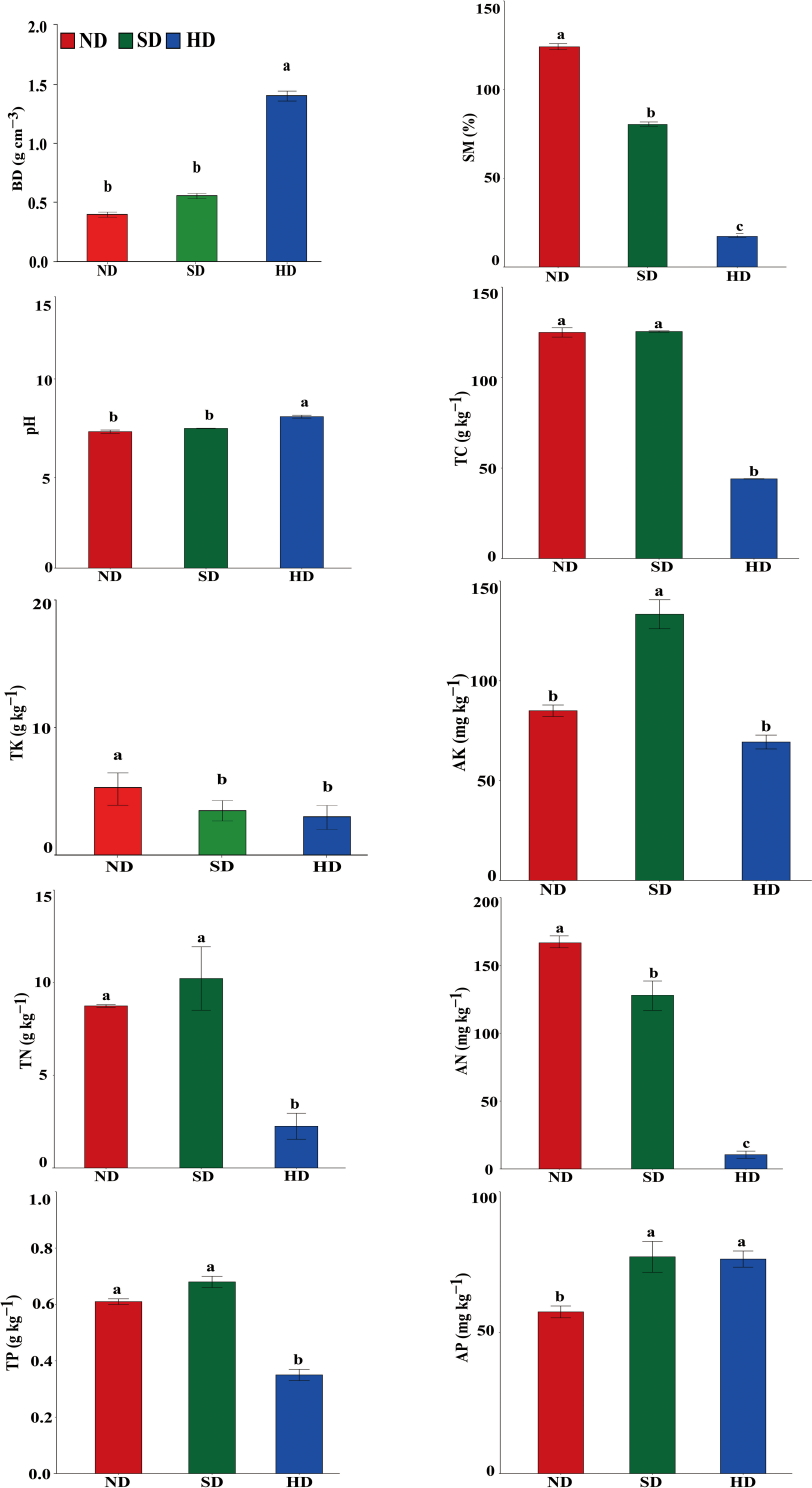
Soil physicochemical properties of the non-degraded (ND), slightly degraded (SD) and heavily degraded (HD) alpine wetland areas. Note: mean ± standard error (n = 3). Values within the same line followed by the same letter do not differ at *P* < 0.05. BD, bulk density; SM, soil moisture content; TC, total carbon; TN, total nitrogen; TP, total phosphorus; TK, total potassium; AN, available nitrogen; AP, available phosphorus; AK, available potassium.

There were significant differences in soil physicochemical properties within the different degraded areas of alpine wetlands (**Figure 2**). The pH in the HD area was significantly higher than that in ND and HD areas, reaching 8.37 ± 0.13. TC, TN and TP in ND area was not different to SD area, but was significantly higher than in HD area. There were no significant differences in TK between the SD and HD areas, but between ND and SD/HD there were. The SM and AN significantly decreased with increasing degradation degree, while the BD was significantly increased with degradation level. AP content was significant different between ND and SD/HD; there was less AP in ND. The AK was significantly higher in SD than in ND/HD.

### Diversity of soil bacterial community in degraded wetlands

**Figure 3:**
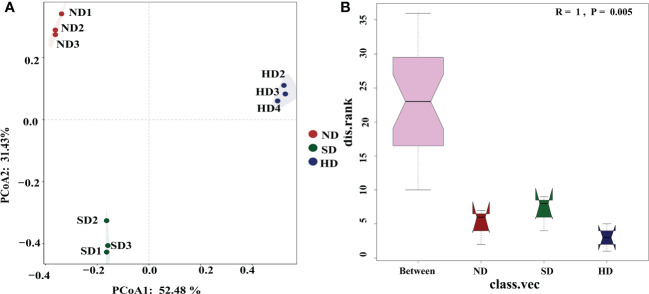
**(A)** Principal coordinate analysis (PCoA) plot of the first two principal components based on soil Bacterial community compositions based on Bray-Curtis distance matrix at each area; **(B)** Bacterial community β-diversity differences; Note: R-value is between (-1,1), R-value > 0, indicating that the difference between groups is significant. R-value = 0, indicating that the difference within the group is greater than the difference between groups. The reliability of statistical analysis is expressed by P-value, and *P* < 0.05 indicates that the statistics are significant.

The library coverage of all of the sequencing samples was higher than 0.99, which was sufficient to detect most bacteria (**Table 3**). Rarefaction curves reached a plateau trend for all samples and showed that sequencing depth was sufficient to cover the microbial diversity in all samples (**Figure S1**). After the chimeras were removed, the number of OTUs was 968-1346; after removing unclassified OTUs at domain level, chloroplast and mitochondria, the final number of OTUs was 963-1341. There were no differences in ACE, Chao1, and Simpson indexes among the degraded alpine wetlands. The Shannon index was the lowest in the ND area, and significant different to SD/HD (**Table 3**). Based on the result of PCoA (**Figure 3A**), the bacterial communities showed differences among the different degradation areas, and the ANOSIM showed that degradation had a significant effect (R=1, P=0.005) on the bacterial community beta-diversity (**Figure 3B**). Suggesting that there were obvious differences in bacterial communities under the condition of wetland degradation.

**Table 3:** Diversity of the soil bacterial communities of the non-degraded (ND), slightly degraded (SD) and heavily degraded (HD) alpine wetland areas.

	ND	SD	HD
**OTUs**	1051.33 ± 41.96^b^	1253.33 ± 14.75^a^	1238.33 ± 58.05^a^
**Library coverage (%)**	99 ± 0.00 ^a^	99 ± 0.00 ^a^	100 ± 0.00 ^a^
**ACE index**	1332.68 ± 49.58^a^	1487.40 ± 15.91^a^	1436.87 ± 65.09^a^
**Chao1 index**	1372.74 ± 57.67^a^	1510.97 ± 15.79^a^	1489.6 ± 62.64^a^
**Simpson index**	0.01 ± 0.0009^a^	0.01 ± 0.0014^a^	0.01 ± 0.0004^a^
**Shannon index**	5.42 ± 0.07^b^	5.67 ± 0.05^a^	5.65 ± 0.09^a^

mean ± standard error (n = 3). The same lowercase letter indicates no significant difference, but the difference is significant at the 0.05 level.

### Differences of wetlands soil bacterial community composition under different degradation

**Figure 4:**
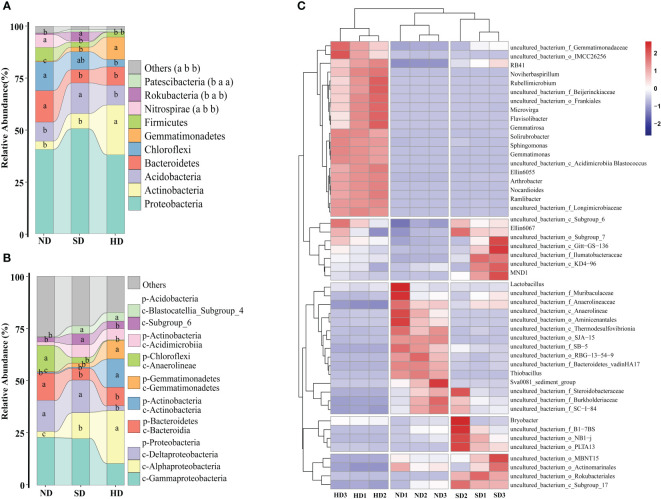
Bacterial community composition of the non-degraded (ND), slightly degraded (SD), and heavily degraded (HD) alpine wetland areas. Mean ± standard error (n = 3). **(A)** Bacterial phyla (p), **(B)** classes (c), **(C)** classified genera (top 50). In the stacked diagram, the same lowercase letters indicate no significant difference.

At phylum level, the Proteobacteria (ND: 40.62%; SD: 50.61%; HD: 38.03%) was the dominant phylum among all samples (**Figure 4A**). The second most dominant phylum of the bacterial communities was Actinobacteria (ND: 3.95%; SD: 7.27%; HD: 23.97%). The other dominated phyla were Acidobacteria (ND: 9.20%; SD: 14.80%; HD: 9.55%), Bacteroidetes (ND: 15.17%; SD: 16.37%; HD: 8.87%), Chloroflexi (ND: 13.92%; SD: 8.68%; HD: 3.55%). The relative abundance of Gemmatimonadetes in the HD area was significantly higher than that in ND and SD areas (*P* < 0.05). There were no significant differences in relative abundance of Nitrospirae between the SD and HD areas, but between ND and SD/HD there were (*P* < 0.05).

At class level, Gammaproteobacteria (ND: 22.85%; SD: 22.53%; HD: 10.29%) and Alphaproteobacteria (ND: 2.83%; SD: 12.38%; HD: 25.36%) were the most abundant in the soil of the different degraded wetlands, followed by Deltaproteobacteria, Bacteroidetes, Actinobacteria, Gemmatimonadetes, Anaerolineae, and others (**Figure 4B**). The abundances of Alphaproteobacteria, Actinobacteria, and Gemmatimonadetes significantly increased with degradation degree. **Figure 4C** shows the relative abundance of the top 50 bacteria genera in the nine wetland samples and that there were significant differences among soil bacterial genera in the different degraded areas, there are specific communities for HD, ND and SD. There are 20 specific communities with higher relative abundance in HD area, including Sphingomonas, Gemmatimonas and Gemmatirosa etc. Similarly, there are 15 in the ND area (uncultured_bacterium_f_anaerolineae, uncultured_bacterium_o_SJA-15 etc.), and 15 in SD area (uncultured_bacterium_o_Rokubacteria, uncultured_bacterium_c_Subgroup_17 etc.).

**Figure 5:**
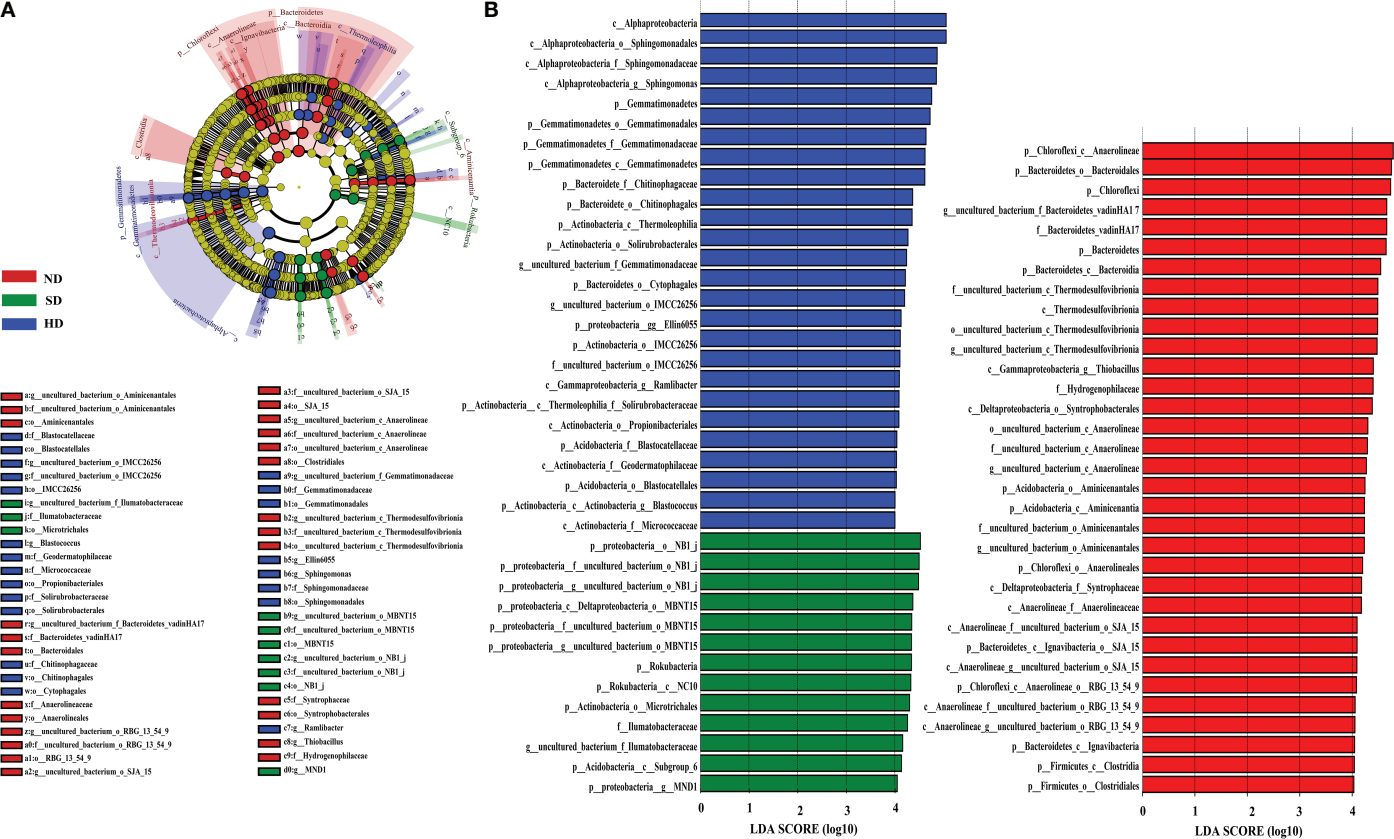
LEfSe analysis of the Bacterial abundance in the non-degraded (ND), slightly degraded (SD), and heavily degraded (HD) alpine wetland areas. **(A)** Phylogenetic tree with the ND, SD and HD bacterial biomarkers obtained with LEfSe analysis. **(B)** Histogram of the LDA scores computed for the differentially abundant bacteriaamong the degraded alpine wetlands identified with a threshold value of 4.0. Note: The LEfSe analysis was performed at the phylum to genus levels.

LEfSe analysis showed that when the LDA threshold was 4.0, there were 72 bacterial taxa (from phylum to genus level) with statistically significant differences among different degradation degrees (**Figure 5**). In the ND area, 33 biomarkers were found, whereas the SD and HD areas had 13 and 26 biomarkers, respectively. Anaerolineae and Bacteroidales were the ND biomarkers with highest relative abundance. uncultured deltaproteobacteria (NB1-j) was the SD biomarker with highest relative abundance. Alphaproteobacteria was the HD biomarker with highest relative abundance, followed by Sphingomonadales, *Sphingomonadaceae*, *Sphingomonas* and Gemmatimonadetes.

### Relationship between the changes in bacterial community and environmental factors

**Figure 6:**
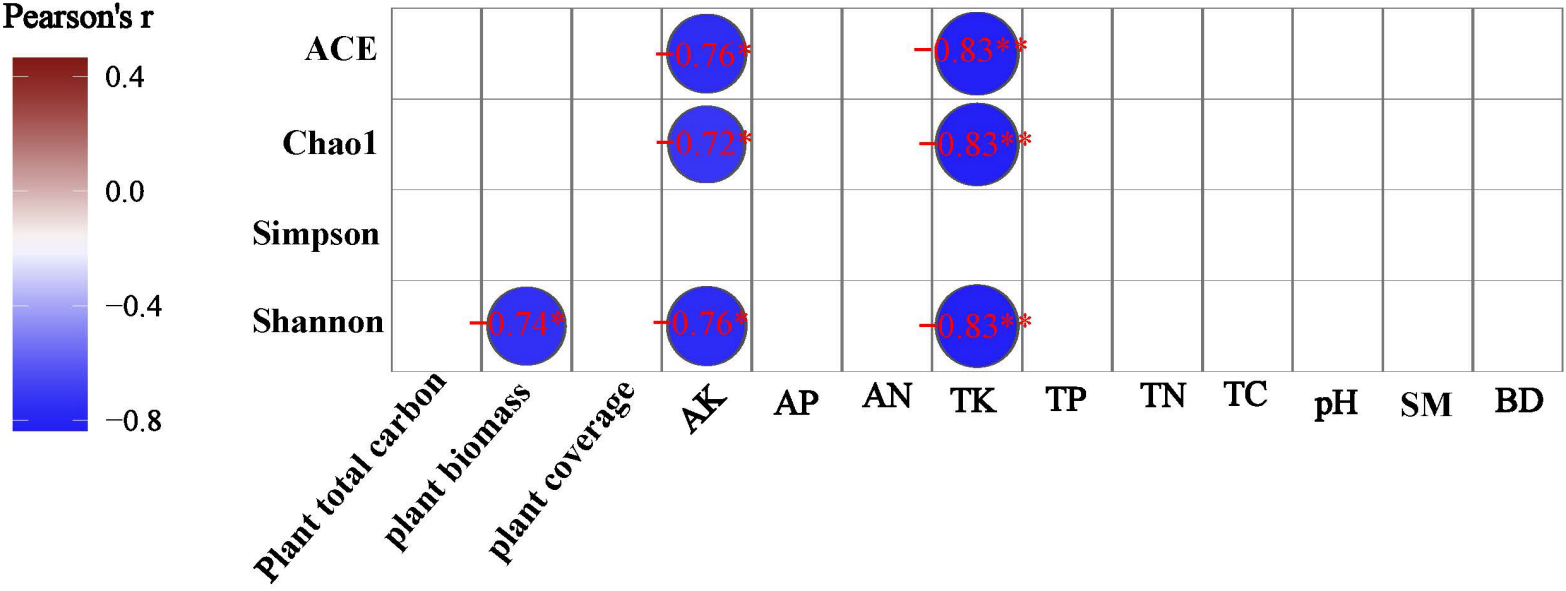
Pearson correlation analysis between alpha-diversity indices and environmental factors.

A Pearson correlation analysis was made between environmental factors and the bacterial community alpha diversity indices under different degrees of degraded wetlands. TK and AK were negatively significantly correlated to ACE, Chao1 and Shonnon index, and above-ground biomass was negatively significantly correlated to shannon diversity index (**Figure 6**).

**Figure 7:**
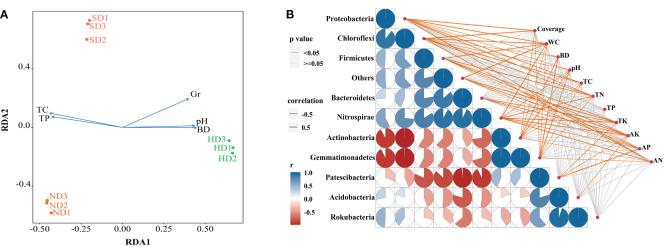
Correlation analysis between the bacterial community structure and environmental factors in the non-degraded (ND), slightly degraded (SD), and heavily degraded (HD) alpine wetland areas. **(A)** Distance-based redundancy analysis (db-RDA) of the soil bacterial community with environmental factors. **(B)** Spearman correlation analysis between the dominant bacteria phyla and environmental factors. Pairwise comparisons of the environmental factors are shown with a color gradient denoting the Spearman correlation coefficient. Taxonomic bacteria (based on the phylum level) community composition is related to each environmental factor by partial (geographic distance-corrected) Mantel tests. The edge width corresponds to the Mantel’s r statistic for the corresponding distance correlations. The edge color denotes the statistical significance based on 9,999 permutations. Note: n = 3. Coverage: total plan coverage; SM: soil moisture content; BD: bulk density; TC: total carbon; TN: total nitrogen; TP: total phosphorus; TK: total potassium; AK: available potassium; AP: available phosphorus; AN: available nitrogen.

The results of db-RDA showed that the samples in each group were close, and the groups were distant **Figure 7A**. Bacterial community structure was significantly correlated with TC and Gr, among which TC had the most significant (*P* < 0.01) influence on bacterial community structure (**Table 4**). A spearman correlation analysis was made between environmental factors and the bacterial community composition (at phylum level) under different degrees of degraded wetlands, and showed a significant correlation among the dominant phyla (**Figure 7B**). The results showed a significant correlation between the dominant phyla. The relationships between Gemmatimonadetes and Actinobacteria, Gemmatimonadetes and Chloroflexi, Actinobacteria and Chloroflexi, and Rokubacteria and Acidobacteria were the most significant ones. Relative abundance of Proteobacteria and Chloroflexi were significantly positively correlated with plant coverage, SM, TC and TN; and Acidobacteria was significantly negatively correlated with AK.

**Table 4:** Effects of environmental factors on bacterial community structure in non-degraded (ND), slightly degraded (SD) and heavily degraded (HD) alpine wetland areas.

Indices	Df	Variance	F	Pr(>F)	Significance
TC	1	0.2660	11.8336	0.003	**
TP	1	0.0257	1.1413	0.372	
pH	1	0.0611	2.7205	0.063	
BD	1	0.0294	1.3099	0.341	
Gr	1	0.0810	3.6019	0.042	*

*: P<0.05; **: P<0.01. TC, total carbon; TP, total phosphorus; BD, bulk density; Gr, Gramineae.

### Soil bacterial community functions under different degraded wetlands

**Figure 8:**
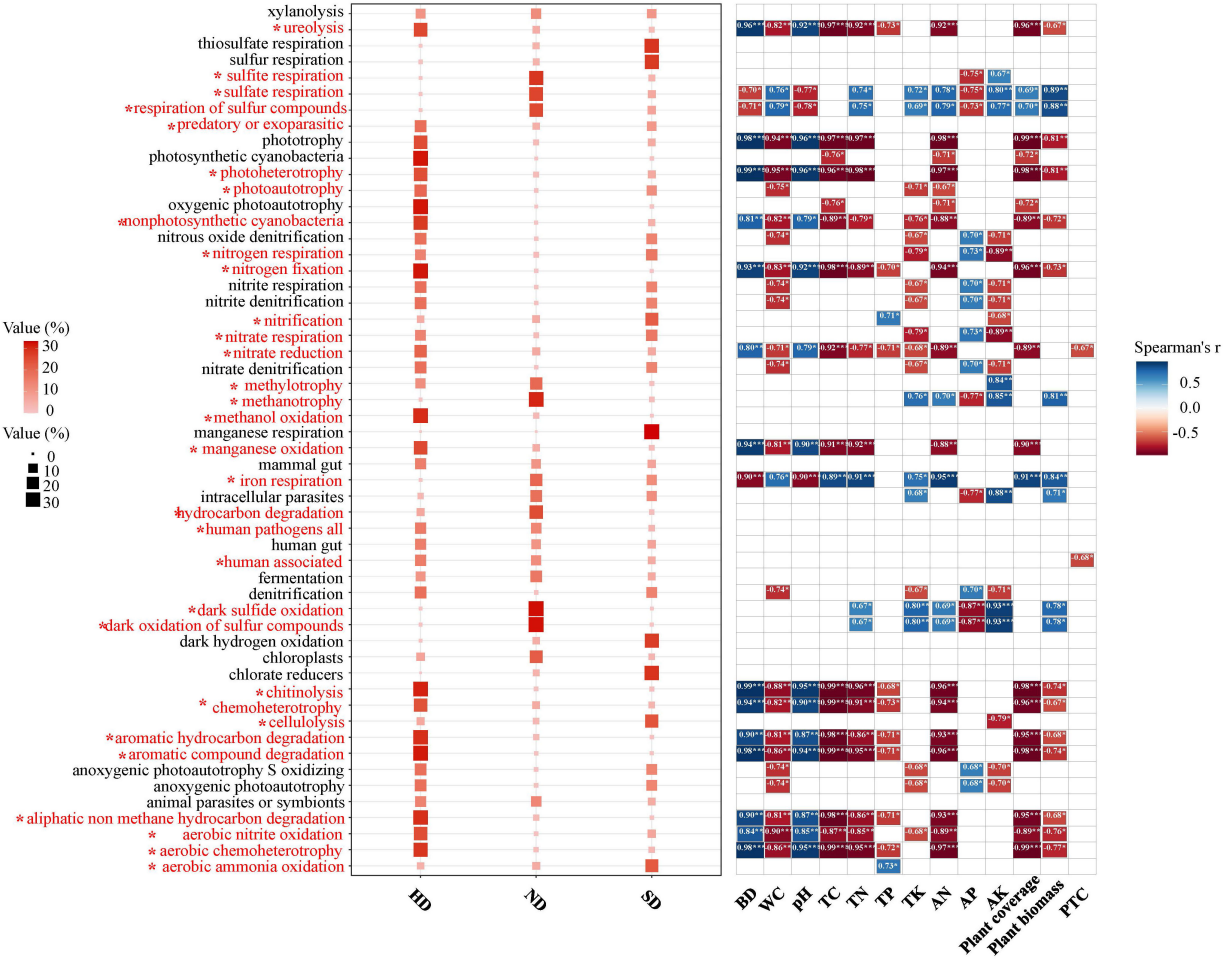
Bacterial community functions of the non-degraded (ND), slightly degraded (SD), and heavily degraded (HD) alpine wetland areas. Functional Prediction of Bacterial Communities; Spearman correlation analysis between community function and environmental factors; *: *P*<0.05; **: *P*<0.01; ***P<0.001.

A total of 54 function were observed by the FAPROTAX functional annotation (**Figure 8**). Through the analysis of variance, it was found that 32 of the 54 functional pathways had significant differences in different degrees of degradation. The chemoheterotrophy process is the main bacterial metabolic process in Bayinbuluk alpine wetland, followed by aerobic chemoheterotrophy, fermentation, nitrification, aerobic ammonia oxidation. The above five processes exist in all degraded areas (ND, SD, HD), indicating that soil bacterial in the different degraded area of Bayinbuluk alpine wetland actively participate in the biogeochemical cycle of elements such as C, N, H and S. As can be seen from **Figure 8**, with the intensification of degradation, the bacterial-dominated fermentation process and most of the sulfur-related functional processes weaken, but its relative abundance is not significantly different. In HD area, chemoheterotrophy, aerobic chemoheterotrophy, ureolysis and chitinolysis are significantly enhanced (*P* < 0.05). SD area showed the highest nitrification and aerobia ammonia oxidation. From the correlation analysis between environmental factors and bacterial community function, 39 functional pathways were significantly affected by environmental factors (**Figure 8**). SM was significantly negatively correlated with majority functional pathways, followed by soil TC.

## Discussion

### Response of environmental factors to alpine wetlands

Plant community structure can directly reflect the degree of degradation (Hirsch et al., 2016). In our study, the Cyperaceae proportion and the total vegetation coverage decreased significantly with the intensifying degradation. This result is consistent with the research results of alpine grassland degradation in Qinghai-Tibet Plateau. With the aggravation of grassland degradation, the total vegetation coverage and Cyperaceae coverage decreased from 94.00% and 41.33% to 24.60% and 0.00% respectively (Zhou et al., 2019). The reason may be that Cyperaceae plants have developed roots and can fully absorb water and nutrients to adapt to wetland environment. However, due to the aggravation of wetland degradation, the soil moisture and nutrient content decrease, which is not favorable for Cyperaceae growth, and is gradually replaced by other grasses (Wang et al., 2018). At the same time, overgrazing (cattle, sheep, horses, etc.) is an important reason to change the physical and chemical properties of wetland plant communities and soil. Specifically, the trampling and nibbling behaviors of animals could explain the changes in the plant community and in the physicochemical properties of wetlands. Chu et al. (2016) reported that increasing soil bulk density will limit the movement of nutrients and water in the soil, and directly affect the absorption of nutrients by plants. In addition, research shows that the succession pattern of alpine meadow plant community changes with the aggravation of degradation degree, and the dominance of the plants of Spartinae and Cyperaceae will decrease, as observed in the studied area (Liu et al., 2018). Similarly, in our study, it was found that the dominance of Cyperaceae plants decreased in SD area, but did not exist in HD area. However, the rhizomatous grass (Leymus chinensis), which is more drought-tolerant, has a greater advantage in the competition (Li et al., 2017) and becomes the dominant species in HD area. At the same time, the difference of plant community structure changes the input of litter, thus affecting the nutrient cycle, changing the soil fertility and further affecting the bacterial community structure (Pereira et al., 2021). Therefore, there are direct and indirect relationships between the characteristics of plant communities and the structure of soil bacterial communities.

The soil physicochemical properties are the key factors that affect the soil bacterial community structure (Gu et al., 2018). Grazing affects the surface soil, gas emissions, and water conduction, increases the surface exposure, and enhances surface transpiration, which leads to reduced soil water content and eventually aggravates soil degradation (Cheng et al., 2016). A reduction of SM is the most direct response to alpine wetland degradation (Lin et al., 2019). Grazing affects the nutrients cycling in the ecosystem and changes the soil chemical structure by increasing the stomping, feeding, and excrement of livestock (Xiong et al., 2015). Previous studies have reported that nitrogen is the main factor that determines the primary productivity of plants. In our study, with the increase of grazing intensity, TN and AN content in HD area were significantly lower than those in SD and ND areas (**Figure 2**). This may be because grazing increases the ratio of soil C to N, which leads to nitrogen mineralization (Wang et al., 2014). At the same time, our research found that SM, TC and other soil physical and chemical factors changed significantly with the increase of degradation degree. These results are similar to the research results of Zoige Wetland (Gu et al., 2018). The contents of TC and AN decreased significantly with the increase of degradation degree, while the content of available potassium increased first and then decreased (Gu et al., 2018). Most of the K ingested from animals and plants returns to the soil in the form of AK through excrement, which makes the AK content in SD area higher than that in ND area (Su et al., 2015). In addition, this study found that with the aggravation of degradation, the content of AP increased, while the content of TP decreased, mainly because animals excreted P back to the soil (Wang et al., 2022), which led to the increase of AP. Furthermore, the SM content will decrease after alpine wetland degradation, which affects the SOC (Lin et al., 2019) and ultimately the global C cycle process (Xu et al., 2016). Soil nutrients, including N, may directly or indirectly affect the diversity and structure of soil bacterial communities by affecting plant communities (Gu et al., 2018).

### Response of bacterial community to alpine wetlands degradation

In our research, degradation has significant effects on both Shannon index and β-diversity (*P* < 0.05). It was similar to those of Zhou et al. (2019) on degraded alpine grassland, who showed no significant variations in the Chao1 and Shannon indexes among the degraded alpine steppes. Previous studies have found that the change of water conditions caused by wetland degradation has an important impact on soil bacterial community diversity (Mentzer et al., 2006). Wu et al., 2021a found that compared with meadow soil and sandy soil with low water content, the Shannon index of swamp soil with high water content is obviously lower. Too high or too low soil water content is not conducive to the survival and reproduction of bacterial communities (Moche et al., 2015). Therefore, the low shannon index in ND area in this study may be due to the high-water content. SD area is a transitional zone of alpine wetland degradation and a special habitat with frequent changes of groundwater level. The results of PCoA analysis and ANOSIM analysis (**Figures 6A** **, B**) showed that there were significant differences in the structure of soil bacterial communities in different degradation areas (R=1, P=0.005). It should be pointed out that although degradation has no significant effect on community richness, it does not mean that there is no significant difference in soil bacterial community structure in different degraded areas.

The bacterial community composition changed significantly with increasing alpine wetland deterioration. Proteobacteria was the dominant phylum in the degraded Bayinbuluk alpine wetland. Proteobacteria widely exist in alpine ecosystems, participate in biogeochemical processes, and have strong survival and adaptability. Although, Proteobacteria was the most abundant phylum in all the different studied areas, there were no significant differences among them. The other dominant phyla were Actinobacteria, Acidobacteria, Bacteroidetes, Chloroflexi, and others. Actinobacteria have strong metabolic and repair functions at low temperatures (Johnson et al., 2007). Studies have shown that Actinobacteria predominate in the dry and cold regions of the arid McMurdo Valley in Antarctica at high altitudes (Goordial et al., 2016). Goordials’s results confirm that Actinobacteria can adapt to low temperatures, dry environments, and low nutrient content. Actinobacteria are widely found in sandy degraded soils in the Tibet plateau (Gu et al., 2018), which is consistent with the results obtained here, and its highest relative abundance in the HD area (**Figure 4**).

The differences in bacterial composition between alpine wetlands and alpine grasslands may be caused by water differences and they have different vegetation (Zhou et al., 2019; Wu et al., 2021a). Zhou et al. (2019) reported that Actinobacteria was the most abundant in degraded grassland. However, Wu et al. (2021a) indicated Proteobacteria was the dominant phylum in degraded alpine wetland. Wetland degradation affects soil moisture content, and the decrease of soil moisture further affects the average relative abundance of Actinobacteria. The results showed that bacterial community abundance responded to water changes in different ways under drought and non-drought conditions. With the decrease of soil water content, the abundance of Actinobacteria will increase. The reason for this is that Actinobacteria have a thick layer of peptidoglycans and the ability to produce endospores helps them become dominant in long-term dry environments (Nicholson et al., 2000; Fabia and Blair, 2009; Fierer et al., 2012; Hernández et al., 2019; Coban et al., 2022). In our research, these two phyla were significantly lower in HD areas in comparison to ND and SD area (**Figure 4A**). Acidobacteria is terrestrial diderm bacteria (Maestre et al., 2015) and its abundance decreased during drying (Coban et al., 2022). The bacterial communities in alpine regions around the world are similar but there are significant differences in soil bacterial communities with different degradation degrees in different regions. Although soil types and climates are different in different areas, bacterial communities have similar responses to soil moisture changes (Romain et al., 2013). In Qinghai-Tibetan plateau the relative abundance of Bacteroidetes decreased with the decrease of soil moisture (Zhou et al., 2019). Wu et al. (2021a) found the relative abundance of Bacteroidetes were significantly lower in swamp meadow in comparison to meadow wetland. Our research showed, in HD the relative abundance of Bacteroidetes were significantly lower than SD and HD. The relative abundance of Bacteroidetes in this study reached 15.17%-8.87% (**Figure 4A**), which is higher than that reported in Zhou et al. (2019) (3.724%-2.274%). C mineralization are strongly affected Bacteroidetes (Fierer et al., 2007), thus the TC and SM were positively correlated with relative abundance of Bacteroidetes (**Figure 7B**).

LEfSe analysis was used to identify biomarkers of wetlands with different degradation degrees and indicate the difference in soil bacterial community composition (**Figure 5**). 72 bacterial markers showed significant differences when the LDA threshold was 4.0, as shown in **Figure 5**. Alphaproteobacteria was the most abundant biomarker in HD, followed by Sphingomonadales (class: Alphaproteobacteria), Sphingomonadaceae (class: Alphaproteobacteria) and *Sphingomonas* (class: Alphaproteobacteria). Their content increased significantly with increasing degradation. Thus, we used *Sphingomonas* as a biomarker in HD. *Sphingomonas* can survive in extreme environments, because *Sphingomonas* has a special metabolic regulation mechanism to adapt to the changeable environment (especially the nutrient-deficient environment), and can efficiently adjust its own growth to resist many adverse environmental changes (Eguchi et al., 1996; Chen et al., 2004; Hu et al., 2007; Roggo et al., 2013). In addition, we used uncultured deltaproteobacteria (SD) and uncultured bacterium anaerolineae (ND) as biomarkers. In summary, our results indicated that the different degraded areas have their own bacterial communities.

### Relationship between environmental factors and bacterial communities

The main goal of our research is to understand the relationship between environmental factors and soil bacteria. The response mechanism of soil bacterial communities in fragile ecosystems to degradation has always been the focus of global research. The results of db-RDA showed that environmental factors, especially TC and Gr, had significant correlation with the changes of bacterial communities. In addition, there are differences in the characteristics of vegetation communities in different degraded regions of alpine wetlands. The degradation leads to the difference of SM in different degraded regions, and the different water-holding capacities of vegetation communities will also lead to the change of bacterial community structure, especially the influence on aerobic and anaerobic bacterial communities will be amplified. Maestre et al. (2015) showed that aridity indirectly affected bacterial community diversity and abundance by strongly affecting pH, SOC and total plant coverage. TN and TK may indirectly affect soil bacterial community composition by affecting plant growth (Wang et al., 2020). In our study, BD increased significantly with the aggravation of degradation (**Figure 2**), thus directly affecting the survival of soil bacteria by affecting the soil porosity.

Han et al. (2018) found that vegetation coverage may be an important factor affecting the diversity and structure of soil bacterial communities. The Cyperaceae coverage and plant species richness in the HD area were significantly lower than ND and SD areas. In our study, the Proteobacteria, Chloroflexi, Firmicutes, and Nitrospirae are positively correlated with plant coverage. The correlation analysis shows that the vegetation coverage, TC, TN, TP is significantly positively related to Proteobacteria and Chloroflexi, (**Figure 7B**). This indicates that their suitable for living in an environment with sufficient nutrients (Zhou et al., 2019). Proteobacteria can live in a wide range of environments and a good environment is more conducive to Proteobacteria accumulation (Xia et al., 2019; Yang et al., 2020), which is verified by the reduced abundance of Proteobacteria in the HD area compared with the ND and SD areas (**Figure 4**). In addition, there are differences in the viability of bacterial groups at the genus level. Similar to Actinobacteria, Gemmatimonadetes can release spores to resist extreme environment (Genderjahn et al., 2018).

In the studied alpine wetlands, Actinobacteria and Gemmatimonadetes are positively correlated with Bacteroidetes, Nitrospirae and Firmicutes, and negatively correlated with Chloroflexi. The result of correlation analysis showed that the Actinobacteria and Gemmatimonadetes were negatively correlated with soil water content, while the Bacteroidetes, Nitrospirae and Firmicutes were positively correlated. This suggests that when the soil water content decreases, the relative abundance of the former will increase, while the average relative abundance of the latter will decrease. This is because Bacteroidetes and Chloroflexi like anaerobic environments (Klatt et al., 2013), so their abundance will decrease when the water content decreases, which is closely related to their own metabolic function. In addition, Acidobacteria is only significantly correlated with AK. Therefore, Acidobacteria can survive better in areas with higher AK content (Fierer et al., 2007).

Through FAPROTAX function prediction, it was found that there are many bacterial taxa that are involved in different elements biogeochemical cycles, such as C, N and S, in the different degraded areas of the Bayinbuluk alpine wetland. Through the analysis of variance, it was found that with the intensification of wetland degradation, the sulfate respiration process of the bacterial community was significantly weakened. Sulfate respiration was positively correlated to the contents of SM, TN, TK AN, above-ground biomass, vegetation coverage, and AK (**Figure 8**). This means that environmental factors play a key role in the process of sulfate respiration (Ren et al., 2018). In addition, the function of bacterial community is closely related to aboveground plants, because aboveground plants provide different survival materials for bacterial community through litter input and root exudates, which further causes the change of bacterial community function (Tolli and King, 2005). In our research, wetland degradation reduces aerobic heterotrophic and chemical heterotrophic functions. This may be because degradation significantly reduced the aboveground biomass and C input and inhibited the assimilation of carbohydrates by soil bacterial communities (Liang et al., 2019). It shows that bacterial function will be affected by changes in environmental factors mediated by degradation. In this study, there are four key nitrogen metabolism processes: nitrogen fixation, aerobic ammonia oxidation, nitrate denitrification, and denitrification. This can indicate that soil bacterial in different degraded areas of the Bayinbuluk alpine wetland can carry out nitrogen cycling. In HD area, the relative abundance of nitrogen fixation is significantly higher than that of SD and ND, while the relative abundance of aerobic ammonia oxidation is lower than that of SD and ND, which means that the aerobic ammonia oxidation process is affected by HD, resulting in the decrease of TN and AN.

In summary, the results of this study show that there are differences in vegetation community characteristics, soil physicochemical properties, and bacterial community characteristics in different degraded regions of alpine wetlands, and there is a close causal relationship between them. Therefore, the unique bacterial communities in each degraded area can be considered as potential markers of degradation.

## Conclusions

This research investigated the vegetation characteristics, soil physicochemical properties, and bacterial community distribution patterns of degraded alpine wetlands in Bayinbuluk. The results showed that the characteristics of the alpine wetland plant community significantly changed with degradation. The coverage of Cyperaceous decreased with increasing degradation of alpine wetland, whereas that of Gramineae gradually increased. The soil TC, SM, and AN significantly decreased with increasing degradation, whereas the pH, AP and BD significantly increased. There were significant differences in the bacterial community structure and composition in the different degraded areas, but no significant differences were observed in community alpha diversity. LEfSe analysis suggests *Sphingomonas* as HD biomarker. The soil bacterial community was significantly related to the TC and Gr. Degradation significantly affected the functions related to C, N and S cycle in soil bacterial community.

In conclusion, degradation alters the vegetation and soil physicochemical properties characteristics of wetlands. This affects the bacterial community structure and composition. Different methods combined with a multi-omics study can be applied in the future to promote research on alpine wetland interconnection and provide further reference data for the sustainability and restoration of wetland ecosystems.

## Data availability statement

The data presented in the study are deposited in the https://www.ncbi.nlm.nih.gov/, accession number PRJNA813909.

## Author contributions

MA, MC, and ZY designed the study and wrote the manuscript. YH and XZ are responsible for field survey and experimental determination. MA and HJ are responsible for analysed the data. All authors have read and agreed to the published version of the manuscript.

## Supplementary material

Supplementary Figure 1 Observed OTUs of non-degraded (ND), slightly degraded (SD), heavily degraded (HD) alpine wetland.
