# Supplementary figures and images for: Response of soil bacterial community to alpine wetland degradation in arid Central Asia
Source: Front Plant Sci. 2023 Jan 4;13:990597. doi: 10.3389/fpls.2022.990597 (PMC9848402; doi:10.3389/fpls.2022.990597)

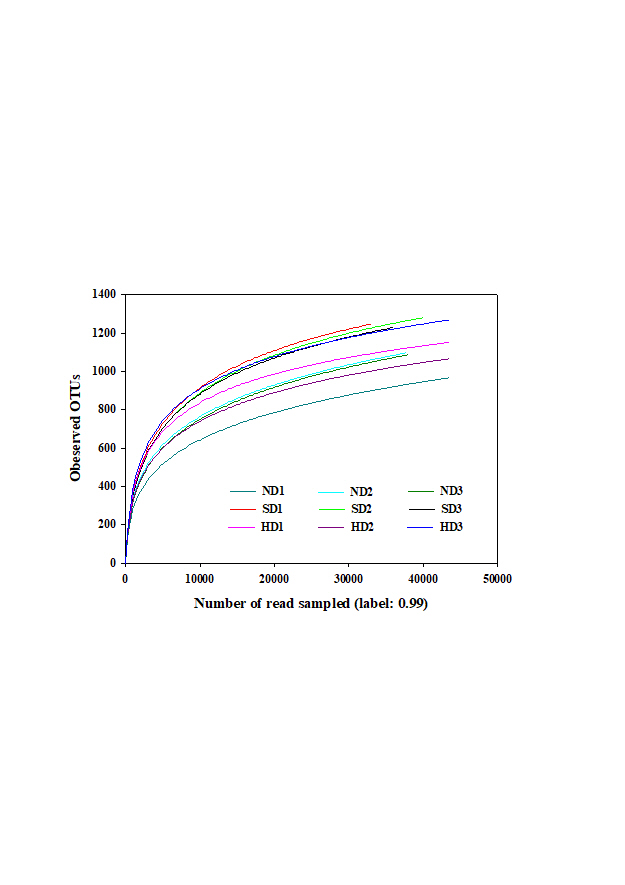

Supplement: Supplementary Figure 1 — Observed OTUs of non-degraded (ND), slightly degraded (SD), heavily degraded (HD) alpine wetland. [file Image_1.jpeg]
